# Mineral-Fortified and Sodium-Reduced Pimento-Paste-Stuffed Spanish-Style *Manzanilla* Olives

**DOI:** 10.3390/foods15101658

**Published:** 2026-05-09

**Authors:** Antonio López-López, José María Moreno-Baquero, Antonio Garrido-Fernández

**Affiliations:** Instituto de la Grasa (IG), Spanish National Research Council (CSIC), Campus Universitario Pablo de Olavide, Edificio 46, Ctra. Utrera km 1, 41013 Sevilla, Spain; jose.moreno.baquero@gmail.com (J.M.M.-B.); agarrido@ig.csic.es (A.G.-F.)

**Keywords:** pimento-paste-stuffed olives, mineral fortification, potassium, calcium, magnesium, nutritional labelling

## Abstract

This study evaluates the concentrations and distributions of major minerals in pimento-paste-stuffed Spanish-style olives, their mineral-fortified products, and their components. Mineral partitioning was assessed using the distribution coefficient (*K_d_*). Model formulations and optimisation were performed using Response Surface Methodology (RSM). In the raw pimento-paste-stuffed olives, sodium was highest in the brine compared to the whole product, pitted olives, or stuffing. The *K_d_* was < 1 on a whole-product basis but approached equilibrium (*K_d_* = 1) when moisture was factored in, indicating a balance between product moisture and brine. Desalting reduced mainly sodium, potassium, and magnesium, followed by calcium, phosphorus, and other minor naturally occurring minerals. Fortification through packaging brines resulted in a product with low sodium (~7.1 g/kg, 30% of the Daily Reference Intake, DRI) and potentially high levels of potassium (up to~5.4 g/kg; 27% DRI), calcium (~4.6 g/kg; 58% DRI), and magnesium (~1.8 g/kg; 32% DRI). For practical purposes, mineral profiles were grouped into four formulation categories: sodium-dominant, calcium-rich, potassium–calcium-abundant, and potassium–magnesium-rich. RSM optimisation showed that brines containing 1.189% KCl, 0.334% CaCl_2_, and 0.978% MgCl_2_ achieved the most desirable mineral balance, enabling substantial sodium reduction while enhancing nutritional value.

## 1. Introduction

Table olives are among the most important fermented vegetable products, with global production reaching about 3.3 million tons in the 2024/25 season [1]. Spanish, Greek, and Californian styles dominate, with Spanish the most popular due to its wide variety of presentations: whole, pitted, stuffed, and sliced. Automatic stuffing machines and a variety of fillings, from pimento gel strips to different pastes, have greatly expanded the options available.

In the 2023/2024 season, Spain produced a total of 407,380 tons, accounting for 15% of the world’s output, including 219,600 tons of green Spanish-style olives and 183,800 tons of Californian-style olives. *Hojiblanca* was the most common cultivar at 48%, followed by *Manzanilla* at 25%, *Cacereña* at 15%, *Gordal* at 5%, and *Carrasqueña* at 2%. About 68% of production is exported, with nearly half of it lye-treated Spanish-style olives [2].

The mineral content (mg/kg olive flesh) in commercial Spanish-style olives varies widely: sodium, 14,378–18,144; potassium, 333–539; calcium, 476–850; magnesium, 99–147; and phosphorus, 69–118. Iron, copper, and zinc are present in the range of 1–6 mg/kg of flesh [3]. Sodium clearly dominates, followed by the other minerals, which are below the recommended Daily Reference Intake (DRI).

Green Spanish-style stuffed olives are typically preserved by pasteurisation [4]. Regulations specify a pH ≤ 4.3, while minimum sodium chloride and lactic acid levels are set in accordance with Good Manufacturing Practices [5]. Since preservation is based on heat treatment rather than salt concentration, a reduction in sodium chloride would be expected. However, in practice, these table olives still contain relatively high amounts of sodium.

A recent survey by the Food and Nutrient Database for Dietary Studies reports the following mineral contents in stuffed olives (in mg/kg of olive flesh): sodium 16,200, potassium 430, calcium 1210, magnesium 120, phosphorus 70, iron 3.1, copper 1.1, and zinc 0.9 [6]. Furthermore, sodium concentrations remain elevated across different stuffing materials. Depending on the filling, sodium levels can increase further (in mg/kg of olive flesh): pepper, 19,300; almond, 17,100; garlic, 14,700; and anchovy, 20,700 [7]. These values indicate that the sodium content in stuffed olives is consistently high and may even increase depending on the filling.

High sodium levels have regulatory implications. In the US, adults consume about 3400 mg/day, compared with the recommended 2300 mg/day for adults ages 14 and above [8]. The FDA’s draft Voluntary Sodium Reduction Goal for olives with additions (Appendix A Table 1, Food Category ID 32) sets a baseline of 18,020 mg/kg, with a target of 16,500 mg/kg within three years and a maximum of 21,700 mg/kg [9]. Canada’s voluntary targets for shelf-stable olives are similar, at 18,700 mg/kg of flesh [10]. In the EU, sodium-reduction strategies under the Farm to Fork Strategy primarily focused on bread and had limited impact on olives due to their relatively low consumption in non-producer countries [11,12].

Recently, the WHO Global Report on Sodium Intake Reduction [13] has reaffirmed that reducing sodium intake plays a key role in protecting populations from the burden of non-communicable diseases, particularly cardiovascular disease, which remains the leading cause of death and disability worldwide. Reducing sodium intake is one of the most cost–effective strategies to improve public health, as it can prevent millions of deaths each year at relatively low implementation costs. Despite the commitment made by WHO Member States to reducing population sodium intake by 30%, progress has been slow, and no country has achieved this target by 2023. In this context, WHO has promoted, within the framework of Food Systems for Health, the reformulation of food and beverage products as an effective global policy for a healthier diet. An analysis of studies from Europe and the United States showed population-wide daily salt intake after reformulation was reduced by 0.57 g compared to pre-reformulation levels, indicating that salt reduction strategies are generally well accepted by consumers [14].

In this context, given the popularity of pimento-stuffed Spanish-style olives, reducing sodium while increasing beneficial minerals is an important goal. Studies on *Manzanilla* (plain) and *Aloreña de Málaga* olives showed sodium reductions of over 50% with potassium, calcium, and magnesium chloride fortification [15,16]. However, research on producing mineral-rich stuffed olives is lacking.

To address this gap, the present study follows a recently developed approach aimed at reducing salt content in table olives during packaging rather than during fermentation, thereby avoiding the risks associated with low salt levels during fermentation and subsequent storage prior to conditioning. For this purpose, fermented olives were first desalted and then packaged in a brine containing a mixture of mineral nutrients salts (KCl, CaCl_2_, MgCl_2_), intended to replace and potentially compensate for the mineral losses occurring during conditioning or normal packaging. To preserve the traditional quality of the product, sodium chloride was partially replaced by equivalent concentrations of these alternative salts. The experimental design was structured to develop a predictive function for estimating the final concentrations of the added salts, using Response Surface Methodology to model their effects. This approach enables the evaluation of mineral distribution between olive and brine, as well as the prediction of optimal brine formulations that enhance nutritional value, maintain consumer acceptance, and meet labelling requirements.

## 2. Materials and Methods

### 2.1. Olives

The *Manzanilla* green olives were processed using the Spanish method, and then stuffed with pimento-paste. The product (30 kg), supplied by JOLCA SCA (Huelva, Sevilla, Spain) in a brine with approximately 7.5% salt, was stored in a cold room (8 ± 1 °C) at the pilot plant facilities of Instituto de la Grasa until use. The olives used in the study averaged 282 fruits per kilogram, corresponding to individual weights of 3.01–3.30 g. In the stuffed product, the proportion of olive flesh to pimento-paste was 84.23:15.77. The moisture contents were 68.49% (*w*/*w*) in the olive pulp and 86.90% (*w*/*w*) in the stuffing, yielding an overall moisture content of 72.96% (*w*/*w*) in the stuffed olives. The density of the filling material was 1.49 g/mL, whereas the pit density was 1.39 g/mL. The average density of the stuffed olives was 0.999 g/mL, slightly lower than that of the unprocessed olives (pulp plus pit), which averaged 1.03 g/mL [17]. Given the comparable densities and proportions, the stuffing process can be interpreted as replacing the pit with the filling material.

### 2.2. Experimental Design and Packaging

Before packaging, the stuffed olives were desalinated in a cold room (8 ± 1 °C) until the pulp reached 2.5% NaCl. This level was achieved by adjusting the olive-to-water ratio to the equilibrium level. The desalted olives were then packaged in glass jars (170 g olives/130 mL brine) containing different brine formulations (Table 1).

These brines consisted of mixtures of KCl, MgCl_2_, and CaCl_2_, with the total chloride concentration constrained to 25 g/L (the expected level). The experimental design was generated using Design-Expert 13.0 (Stat-Ease Inc., Minneapolis, MN, USA). Since the actual concentrations depend on hydration level and olive/brine ratio, the proportions were corrected accordingly. All brines also contained 2.5% NaCl to maintain flesh sodium levels after equilibrium, resulting in a final brine concentration of 5% total salts, as recommended for lye-treated green table olives [5]. Lactic acid was added to achieve a concentration of 0.5% (*w*/*v*) and adjust the pH to around 4.0. Finally, the jars were pasteurised at 85 °C for 8.5 min to reach a pasteurisation unit (PU) of ${PU}_{62.4\;{}^{\circ}C}^{5.25}\;\geq$ 15, simulating the industry’s current stabilisation process. The packaged olives were stored at 20 ± 2 °C in the Instituto de la Grasa (Sevilla, Spain) facilities for two months to allow for equilibrium and simulate shelf life.

### 2.3. Determination of Minerals in the Flesh and Brines

All glassware was treated with 6 mol/L HCl overnight, then rinsed with distilled, deionised water before use. Mineral determination was performed by calcination, wet mineralization, and direct brine analysis. For calcination, 5 g of homogenised olive pulp was weighed (analytical balance GR-120, A&D Instrumentals Ltd., Abingdon, UK) in a quartz capsule and calcined at 550 °C for 8–10 h in a furnace (L-9/11-B-180, Nabertherm GmbH, Lilienthal, Germany). The ashes were dissolved in 6 mol/L ultra-pure HCl, filtered into a 25 mL volumetric flask, and topped up to volume with deionised water; blanks were prepared in parallel.

Wet mineralisation was performed following the procedure outlined by Mesías et al. [18]. Olive flesh (2.5 g) or brine (20–25 mL) was digested in Pyrex tubes with 65% HNO_3_, then with HNO_3_ (65%): HClO_4_ (60%) (1:4) until the solution became clear. After cooling, the digests were transferred to 25 mL volumetric flasks and diluted with double-deionised water (Sigma, S. Louisa, MO, USA). For direct analysis, the brines were filtered through a 0.2 µm membrane and analysed without further treatment. Blanks were prepared in parallel.

Mineral determination was carried out using atomic absorption spectrometry (GBC 932 AA, Victoria, Australia) with an air–acetylene flame and hollow multi-element cathode lamps (GBC, Photron, Victoria, Australia). Lanthanum (La^+3^) (2000–5000 µg/mL) was added for calcium (Ca^2+^) and magnesium (Mg^2+^) measurements to prevent interferences; ionisation was avoided in Na and K determinations by adding K or Na (2000 or 1000 µg/mL, respectively).

Phosphorus was measured using the spectrocolorimetric method [19], which relies on phosphate–vanadate–molybdate complex formation [20].

Analyses were performed on three independent samples, each analysed in triplicate, and the value for each sample was calculated as the mean of these three determinations.

Reagents were of analytical grade (Panreac, Barcelona, Spain), with standards from Sigma-Aldrich (St. Louis, MO, USA) and PACISA (Madrid, Spain). Additional details can be found elsewhere [3].

### 2.4. Study of the Equilibrium and Relationship Between Mineral Contents in Brine and Flesh

Mineral distribution was assessed using the pseudo-distribution coefficient (*K_d_*) previously developed for table olives [21]. According to EPA terminology, *K_d_* reflects the ratio of mineral concentration in the flesh to that in the brine at equilibrium [22,23,24,25]. Unlike diffusion-based models [26,27], this empirical coefficient provides a straightforward, practical tool for estimating the brine concentrations required to achieve targeted packaged olive flesh compositions.

### 2.5. Modelling the Effect on the Olive Concentrations and Mineral Contents

The effects of different concentrations of KCl, CaCl_2_, and MgCl_2_ in packaging brines on responses such as firmness, moisture, *K_d_*, mineral content, and DRI contribution were analysed using a special cubic regression model [28]. In canonical (Sheffé) form, it is formulated as

(1) $$R=\beta_{0}+\sum_{i=1}^{n}\beta_{i}x_{i}+\sum_{1\leq i<j}^{n}\beta_{ij}x_{i}x_{j}+\sum_{1\leq i<j<k}^{n}C_{ij}x_{i}x_{j}x_{k}+\in$$

where *x*_1_, *x*_2_, and *x*_3_ are the salt concentrations, *R* the responses, *β*s the coefficients, and ∈ the error term. ANOVA and backward selection (*p* ≤ 0.01 for inclusion; *p* ≥ 0.10 for removal) were applied. Additionally, non-significant linear terms were retained when significant interactions were present, because only hierarchical models are scale-independent and can be expressed in actual units [28]. Models were deemed significant when their *p*-values were <0.05, and the lack-of-fit was insignificant (*p* > 0.05). Responses were visualised as 3D surfaces or contour plots in the simplex. The use of RSM is appropriate in this study, since the mineral concentrations are not independent (the mixture of salts was constrained to 2.5%). Additionally, RSM provides more information than any other design and requires fewer experiments than changing the levels of variables in an independent way [28]. Furthermore, the design allows identification of optimum compositions provided the expected trends of the response variables. Finally, it has been demonstrated to be especially suitable for these types of assays.

### 2.6. Statistical Analysis

Design-Expert v. 13.0 (Stat-Ease, Inc., Minneapolis, MN, USA) was used to design the experiment and analyse the data. Statistica software version 8 (StatSoft Inc., Tulsa, OK, USA) was also used to estimate *K_d_*. Multivariate analysis was performed using XLSTAT v 2017 (Stat-Soft, Paris, France).

## 3. Results

### 3.1. Mineral Content and Its Distribution in the Stored Pimento-Paste-Stuffed Olives

The first step of the study involved assessing the raw material (stored stuffed olives) and examining the effect of desalting. To better understand this effect, the whole product, the pitted olives, and the stuffing material were analysed separately. In the stored stuffed olives, the brine sodium content was significantly higher than that of the product, indicating an unusual distribution of NaCl. As shown in Table 2, the sodium concentration in the whole product was 21.2 g/kg, whereas the brine contained the highest concentration at 29.7 g/kg. Additionally, sodium was unevenly distributed among the stuffed olive components: highest in the stuffing paste (26.5 g/kg), followed by olive flesh (pitted olive) (20.1 g/kg).

Distribution coefficients (*K_d_*) for minerals in stored stuffed olives, their components (pitted olives and pimento paste), and brine (Table 3) were calculated using the mineral concentrations of each substrate and brine (Table 2). Additional values on a moisture basis, detailed as footnotes, are also provided in Table 3.

For the entire product, sodium and potassium showed relatively low *K_d_* values, indicating higher concentrations in the brine than in the stuffed olive. These values were similar in pitted olives but lower in the pimento-paste, suggesting greater retention of these minerals in the stuffing. Magnesium displayed *K_d_* values close to 1, with slightly higher values in stuffing than in olives, indicating a particular affinity for the stuffing material. Phosphorus showed a more balanced distribution between the two components. In contrast, calcium, iron, copper, and zinc had *K_d_* values greater than 1, suggesting a stronger affinity for the whole product. Within the pitted olives, iron, copper, manganese, and zinc exhibited higher *K_d_* values across the entire product and olives, confirming a stronger association with the olive flesh than with the stuffing material. Both whole stuffed olives and the stuffing had a higher calcium content than the pitted olives, although it was consistently lower than the cover storage brine.

When calculated on a moisture basis, *K_d_* values increased. Sodium and potassium approached 1, indicating equilibrium between the moisture of the entire product or its components and the brine. Magnesium showed a *K_d_* > 1 across substrates, indicating a strong association with both olive and stuffing components, as well as some retention in organic matter. Phosphorus displayed a *K_d_* > 1, with the highest value observed for pitted olives, pointing to its major association with the olive pulp. Calcium showed the highest *K_d_* in the stuffing, reflecting its role in paste formation. Other microminerals yielded unrealistically high *K_d_* values, consistent with their predominant association with olive pulp.

### 3.2. Effect of Desalting on the Mineral Content of the Stuffed Olives

The sodium level in the desalting solution was higher (9.9 g/kg) than in the stuffed product (around 7.3 g/kg), although the stuffing pastes still retained more sodium (9.3 g/kg) than the olives (6.8 g/kg). Therefore, the desalting solution at equilibrium had a higher sodium concentration than either of its components, confirming the effective extraction of sodium. Overall, desalting reduced sodium in the stuffed olives by 60–66%, with the stuffing paste still containing higher sodium levels than the pitted olives.

Alongside sodium reduction, other mineral nutrients were also lost. Potassium and magnesium were also unexpectedly higher in the storage brine than in the stuffed olives (Table 2). Initially, they were more abundant in the stuffing paste than in the olives, and this trend persisted after desalting. Potassium losses (64.8%) were similar to those of sodium, with a slightly greater reduction in olives (67.1%) than in the paste (62.0%). Magnesium losses (60.6%) were also substantial, with a similar decline in both parts (olives, 59.5%; pimento-paste, 58.8%). The slightly lower magnesium loss compared to potassium suggests a stronger binding of this divalent cation to components of olive flesh.

Calcium, used in pimento-paste preparation, was higher in the stored stuffed product (2.8 g/kg) than in the brine (1.7 g/kg), and higher in the pimento-paste (3.2 g/kg) than in the olive flesh (1.7 g/kg). This pattern persisted after desalting. Calcium losses during desalting were moderate (45%), greater in the paste (49%) than in the olives (40%), and lower than those of potassium and magnesium, indicating stronger binding to the olive flesh (primarily) and paste matrices.

Phosphorus was also relatively abundant in stuffed olives (115 mg/kg), and its losses were lower than those of calcium (Table 2), consistent with its natural presence in olives. It leached readily from olive flesh (37.3%), but from pimento-paste, the release was even higher (42.0%). Micronutrients such as iron, copper, manganese, and zinc showed minor losses, ranging from 3.6% to 37.5%, likely due to their natural origins.

### 3.3. Mineral Contents in Pimento-Paste-Stuffed Olives and Brine in the Experimental Treatments

After packaging and equilibration, mineral contents were measured in the stuffed olives and their components (pitted olives and pimento-paste). Results are presented on a whole stuffed olive (olive plus pimento-paste) (Table 3) and on a moisture (juice equivalent) (Table 3, last column) basis for each treatment (Table 4). This distinction enables comparison of mineral distribution across substrates, as discussed later.

Potassium, calcium, and magnesium levels were consistently higher in the fortified treatments than in the Control. Potassium ranged from 7259 to 2518 mg/kg pulp, calcium from 6212 to 1513 mg/kg pulp, and magnesium from 2428 to 66.27 mg/kg pulp, demonstrating significant fortification potential (note that the lowest levels of calcium and magnesium occur in treatments without their addition). In contrast, sodium decreased from 21 g/kg flesh in the stored stuffed olives to 7.0 g/kg in the packaged product across all treatments, representing a 66.5% reduction.

Phosphorus and micronutrient levels were only slightly lower than in the desalted olives, suggesting minimal impact from packaging brines.

Overall, the treatments produced mineral-fortified stuffed olives with significantly reduced sodium levels. The connection between mineral contents and the initial salt mixtures will be discussed later.

#### 3.3.1. Effect of the Salt Mixture Packaging on the Mineral Distribution Between Stuffed Olives and Cover Brine

As the pimento-paste represents no more than 20% of the product, and *K_d_* differed only slightly between the whole product and pitted olives for most minerals (Table 3), separate evaluation of components in the experimental treatments would not yield significant insights. Therefore, subsequent analyses consider the entire stuffed olive (olive and pimento-paste).

#### 3.3.2. Mineral Distribution Between Product and Brine in the Experimental Treatments

The mineral concentrations for the entire product (olive plus stuffing) and its moisture content are shown in Table 4 and Table 5, respectively. The *K_d_* values for the experimental treatments are listed in Table 6. Micronutrient levels in the brines of the packaged products were below detection limits and were therefore excluded. Overall, the *K_d_* trends were similar to those observed in stored stuffed olives.

Sodium, potassium, and magnesium had *K_d_* values below 1, indicating higher concentrations in brine than in the entire stuffed olives. When calculated on a moisture basis, *K_d_* values for these elements were near 1, indicating an equilibrium between product moisture and brine, with minimal binding to olive organic compounds (solids). These trends suggest that sodium, potassium, and magnesium do not form stable complexes with the olive components.

In contrast, calcium and phosphorus exhibited *K_d_* values greater than 1, indicating a stronger association with the olive flesh components while still being present in the product’s moisture. When calculated based on moisture, their *K_d_* values became unrealistically high, confirming that these minerals are mostly bound to olive and stuffing components.

Finally, variations in *K_d_* among treatments reflected the type and concentration of mineral salts used in the mixtures, which will be examined in detail below.

#### 3.3.3. Insights into the Distribution of the Mineral Nutrients in the Olive Pulp

Beyond *K_d_*, linear modelling was used to analyse the distribution of key minerals between brine and stuffed olives. In this model, the standardised coefficients *βs* indicate the contribution of brine to the stuffed olive. At the same time, the intercept may reflect an initial excess or deficiency—that is, the mineral in the olives not participating in equilibrium due to binding with stuffed olive organic compounds. The model was applied to the entire product (olive plus stuffing) and to its moisture content (Table 7). Model fit was assessed using adj. R^2^ (accounting for the number of parameters in the equation) and the *p*-value of the ANOVA. The models for sodium, potassium, calcium, and magnesium were all significant (Table 7), regardless of the concentration basis. In all cases, β values were significant and close to 1, with very low standard errors, indicating a strong direct contribution. The slopes for sodium, potassium, and magnesium were below 1 (0.77, 0.74, and 0.78, respectively) when calculated for the entire product, but close to 1 (1.02, 0.98, and 1.05, respectively) when based on moisture, suggesting an equilibrium between brine and product moisture. In contrast, calcium exhibited slopes slightly above 1 for whole olives (1.12) and higher for moisture (1.51), indicating substantial accumulation in the product components.

Intercepts revealed additional insights (Table 7). For sodium, the intercept was significant and nearly identical across both estimation methods (−451 for the product and −462 for moisture), indicating a residual amount likely retained in the olive components from the lye treatment or from stuffing formation. Potassium (−7.43 vs. 15.69) and magnesium (15.51 vs. 22.98) intercepts were low or insignificant, consistent with their primary origin from the added salts. In contrast, the intercept for calcium was positive, high, and significant (517 vs. 687), reflecting both the natural presence in olives and stuffing material and the enhancement by added calcium salts.

#### 3.3.4. Effect of the Salt Mixtures on the Distribution Coefficient

Salt mixtures did not affect the distribution coefficients of sodium, potassium, and phosphorus. This outcome is due to their low concentration in the moisture of the pimento-stuffed olives and their minimal affinity for olive components, which allows for a simple equilibrium since phosphorus is naturally present in the olives and was not added in the assay. Conversely, the distribution coefficients for calcium and magnesium were significantly impacted by the salt mixture concentrations used in packaging (Table 8). Models based on stuffed olives and their moisture are linearly related; therefore, only one, focusing on their moisture content, will be discussed.

*Calcium***.** The calcium model was highly significant (*p*-value = 0.0001) and showed no lack of fit (*p*-value = 0.9114). The explained variances (R^2^ = 0.90409; adjusted R^2^ = 0.7850) were high, with good agreement between the two types, and the precision was strong (13.879), well above the threshold of four. In coded factors—which indicate the expected change in response per unit change in the factor while holding others constant—CaCl_2_ concentration contributed the most (Table 9). The contributions of KCl and MgCl_2_ were similar but much lower. The interactions of these salts with CaCl_2_ decreased the Ca distribution coefficient by competing for similar binding sites on stuffed olive organic compounds. A Variance Inflation Factor (VIF) of around 1.6 indicates low collinearity among factors, as values up to 10 are considered acceptable.

The calcium model in terms of actual components was

(2) $$K_{d\;Ca}=0.85\left[KCl\right]+2.18\left[Ca{Cl}_{2}\right]+0.54\left[MgCl_{2}\right]-1.45\left[KCl\cdot CaCl_{2}\right]+0.19\left[KCl\cdot MgCl_{2\;}\right]-1.23[CaCl_{2}\cdot MgCl_{2}]$$

**Figure 1:**
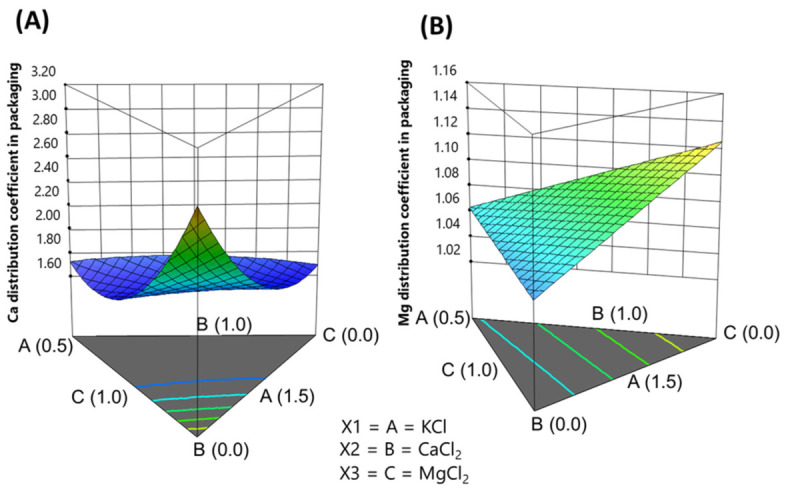
Effect of KCl, CaCl_2_, and MgCl_2_ salt mixtures in the packaging brine on the distribution coefficient of (**A**) calcium and (**B**) magnesium.

As they are adjusted to actual units, interpretation through coefficients should be avoided. Although complex, this function can be easily understood via the simplex plot (Figure 1A). The lowest *K_d_* values are found on a plane between CaCl_2_ concentrations from two-thirds to its maximum level, where, regardless of KCl and MgCl_2_ concentrations, *K_d_* remains nearly at its lowest as CaCl_2_ increases up to the KCl-MgCl_2_ border. This pattern indicates calcium saturation in the stuffed olives. At the highest CaCl_2_ level, excess calcium is only distributed between the brine and the moisture of the stuffed olives but is not further fixed by the olive components. This trend reduces the amount of minerals entering the olives, and the system gradually reaches an equilibrium between the brine and the olive moisture. Conversely, the highest *K_d_* occurs at the CaCl_2_ vertex, where no CaCl_2_ was added. In this case, the natural calcium in the pimento-paste remains firmly in the stuffed olives and does not leach into the brine. This trend keeps calcium levels in the brine at their lowest and *K_d_* at its highest.

*Magnesium*. The magnesium model was significant (*p*-value = 0.0027), with an insignificant lack-of-fit (*p*-value = 0.4969). However, the adjusted variance was acceptable (*p*-value = 0.5966), and precision exceeded the desired threshold of 4, supporting the model’s suitability. In coded factors, MgCl_2_ was the strongest contributor, although KCl and CaCl_2_ also increased magnesium incorporation into the stuffed olives. Very low VIF values indicated that there was no multicollinearity among the factors.

The model equation in actual units was

(3) $$K_{d\;Mg}=0.34\left[KCl\right]+0.34\left[CaCl_{2}\right]+0.28[MgCl_{2}]$$

This linear model creates an inclined plane (Figure 1B) that rises as MgCl_2_ concentration decreases. Without MgCl_2_, the natural magnesium level remains relatively high in the stuffed olives, with little being released into the brine, resulting in a high *K_d_*. CaCl_2_ contributes slightly more than KCl because calcium improves olive texture and reduces magnesium losses to the brine. In contrast, as KCl increases, potassium dissolves in the moisture with minimal impact on magnesium retention in the solids. Consequently, the *K_d_* trend approaches the equilibrium value. However, because potassium retention in stuffed olives is limited, the changes in *K_d_* are also restricted.

### 3.4. Predictions of Mineral Concentrations in Pimento-Paste-Stuffed Olives at Equilibrium Based on Experimental Design Levels

The experimental design enabled the creation of response surfaces to predict the concentrations of the three fortifying minerals in pimento-stuffed *Manzanilla* olives. Model evaluation followed the same systematic process used for distribution coefficients.

*Potassium***.** The linear regression model for potassium was highly significant (*p*-value < 0.0001), with a non-significant lack-of-fit (*p*-value = 0.2329). The explained variance was very high, with close agreement between R^2^ (0.9961) and adjusted R^2^ (0.9954), and excellent precision (signal-to-noise ratio = 88). VIF values were close to one, confirming the absence of collinearity among factors. The coefficient for KCl, in terms of the coded factor (Table 9), was relatively low compared to those for CaCl_2_ and MgCl_2_; however, interpreting the model only from these coefficients may be misleading. To better interpret the model, it was expressed in actual units and represented in the simplex. In actual units, the model equation is

(4) $$K\;in\;pulp\left(\frac{mg}{kg}\right)=3518.97\left[KCl\right]+81.75\left[CaCl_{2}\right]+99.08[MgCl_{2}]$$

**Figure 2:**
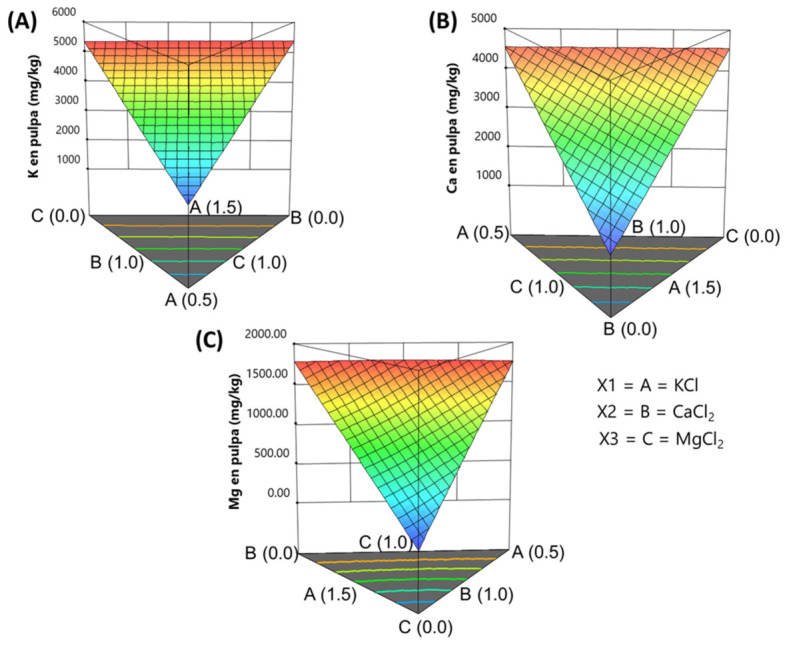
Effect of KCl, CaCl_2_, and MgCl_2_ salt mixtures in the packaging brine on the concentrations of (**A**) potassium, (**B**) calcium, and (**C**) magnesium in fortified pimento-paste-stuffed Spanish-style olives.

The plot in the simplex (Figure 2A) (contour and surface) shows that potassium concentrations gradually increase as one moves from the KCl vertex (lowest KCl content) toward the opposite CaCl_2_-MgCl_2_ edge, with a simultaneous decrease in the concentrations of the other two salts. The highest predicted concentration approaches 4550 mg/kg.

*Calcium*. The regression model for calcium was also highly significant (*p*-value < 0.0001), with a non-significant lack-of-fit (*p*-value = 0.1417). The explained variance was excellent, with R^2^ = 0.9986 and adjusted R^2^ = 0.9984, indicating very close agreement. Model precision was extremely high (148.86 compared to a reference value of 4). The coefficient for CaCl_2_ was lower than that for KCl and MgCl_2_ (Table 9). VIF values were low, indicating no collinearity among the factors.

In actual units, the model was

(5) $$Ca\;\left(\frac{mg}{kg}\right)=458.35\left[KCl\right]+3864.89\left[CaCl_{2}\right]+456.28[MgCl_{2}]$$

The contour plot and surface (Figure 2B) show calcium increases along an inclined plane, with the contour lines running parallel from the CaCl_2_ vertex (no CaCl_2_ added) toward the opposite CaCl_2_-MgCl_2_ side. Calcium concentration increased as CaCl_2_ rose, while decreases in CaCl_2_ and MgCl_2_ co-occurred, indicating that both salts have a similar effect on calcium levels.

*Magnesium***.** The magnesium model was also highly significant (*p*-value < 0.0001), with a non-significant lack-of-fit (*p*-value = 0.1511). The model explained nearly all the variance (R^2^ = 0.9999; adjusted R^2^ = 0.9998). The most influential coefficients in coded units were for KCl, CaCl_2_, and the interaction KCl·CaCl_2_·MgCl_2_, the latter with a negative sign, indicating that this interaction inhibits magnesium absorption in stuffed olives (Table 9). Although the VIF values were higher than those for the previous minerals, they all remained below 2, well under the tolerance limit of 10.

The equation in terms of actual units is

(6) $$Mg\;\left(\frac{mg}{kg}\right)=465.17\left[KCl\right]+1029.19\left[CaCl_{2}\right]+2771.74\left[MgCl_{2}\right]-1115.56\left[KCl\right]\cdot \left[CaCl_{2}\right]-1127.65\left[KCl\right]\;\cdot \left[MgCl_{2}\right]-1739.46\left[CaCl_{2}\right]\cdot \left[MgCl_{2}\right]+1184.86\left[KCl\right]\cdot \left[CaCl_{2}\right]\cdot \left[MgCl_{2}\right]$$

The plot in the simplex (Figure 2C) facilitates easier interpretation. The magnesium content increases as one moves from the MgCl_2_ vertex toward the opposite CaCl_2_-KCl border, with contour lines showing slight curvature due to salt interactions. Within the tested range, magnesium concentrations reached about 2000 mg/kg.

It is important to recognise that the value of these models lies not in their fit but in their predictive ability. They can be used either to estimate the mineral concentrations achievable under specific salt conditions in the packaging brine or to determine the salt combinations required to achieve a targeted mineral profile.

### 3.5. Effect of Fortifying on the Daily Reference Intake (DRI) of Mineral Nutrients

Since most health claims are based on the mineral’s role in the diet, it is useful to compare the salt levels in the packaging brine not only on the mineral content of the stuffed olives but also on their contribution to the Daily Reference Intake (DRI).

*Sodium*. The sodium reduction achieved by replacing NaCl with 2.5% of the fortifying salts was sufficient to significantly lower DRI contributions. In the Control, sodium intake from 100 g of stuffed olives accounted for about 59% of the DRI, whereas in the fortified treatments, this contribution dropped to 29–30%. This reduction of over 25% meets the typical threshold for labelling a food as “reduced in sodium,” allowing the product to be marketed as a healthier option.

Notice that, for DRI models, these values are linear combinations of the actual mineral contents; there is no need to repeat the derivation of the models, so only the model equation and visualisation of the expected contributions will be discussed.

*Potassium***.** Using RSM, the contribution of KCl was predicted as

(7) $$DRI\;K\;\left(\%\right)=+17.59\left[KCl\right]+0.41\left[{CaCl}_{2}\right]+0.50[{MgCl}_{2}]$$

**Figure 3:**
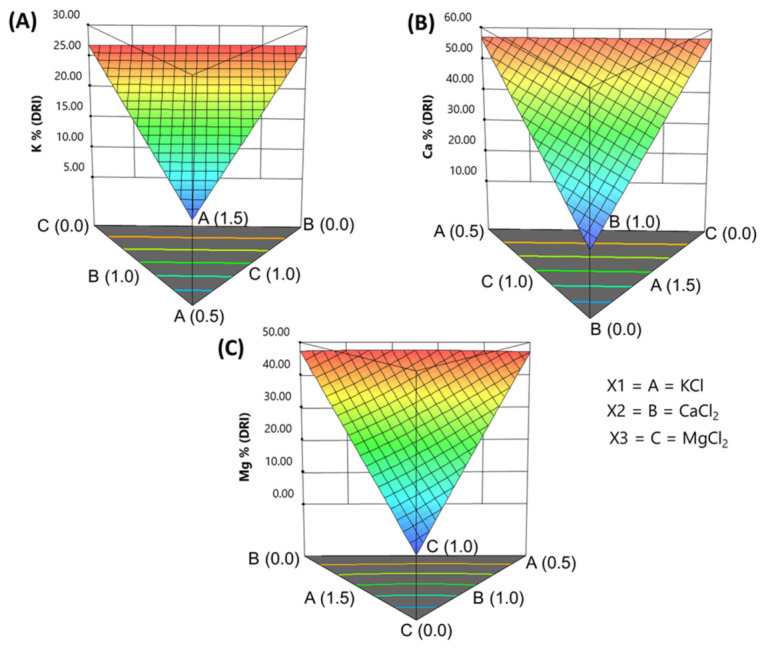
Effect of KCl, CaCl_2_, and MgCl_2_ salt mixtures in the packaging brine on the contribution to % DRI of (**A**) potassium, (**B**) calcium, and (**C**) magnesium in fortified pimento-paste-stuffed Spanish-style olives.

Within the experimental concentration ranges, potassium contribution could reach up to 27% of the DRI. Even at modest KCl levels, pimento-stuffed *Manzanilla* olives could be labelled as a source of potassium (Figure 3A).

*Calcium contribution.* The model equation for calcium contribution was

(8) $$DRI\;Ca\;\left(\%\right)=5.73\left[KCl\right]+48.3\left[{CaCl}_{2}\right]+5.70[{MgCl}_{2}]$$

The CaCl_2_ concentrations in the packaging brine were sufficient to increase calcium contribution to about 55% of the DRI (Figure 3B). This change allows flexibility to adjust formulations, ranging from moderate calcium enrichment to products that can make strong calcium-related claims.

*Magnesium contribution*. The contribution of magnesium (%DRI) was modelled as

(9) $${DRI}_{Mg}(\%)=+12.40\cdot \left[KCl\right]+27.45\cdot \left[{CaCl}_{2}\right]+73.91\cdot \left[{MgCl}_{2}\right]-29.77\cdot \left[KCl\right]\cdot \left[{CaCl}_{2}\right]-30.1\cdot \left[KCl\right]\;\cdot \left[{MgCl}_{2}\right]-46.339\cdot \left[{CaCl}_{2}\right]\left[{MgCl}_{2}\right]+31.59\left[KCl\right]\cdot \left[Ca{Cl}_{2}\right]\cdot \left[{MgCl}_{2}\right]$$

Despite the model’s complexity, which includes several two- and three-way interactions, the simplex representation simplifies interpretation. The response surface is curved, with the highest values along the MgCl_2_ vertex, toward the opposite KCl-CaCl_2_ border, where the maximum contribution occurs. There is a slight, unbalanced decrease as the levels of one salt surpass those of the other. Magnesium contribution reaches up to 50% of the DRI (Figure 3C). Depending on the brine formulation, stuffed olives can therefore be labelled either as “a source of magnesium” or “high in magnesium”.

Overall, these models demonstrated that fortification not only reduces sodium but also substantially increases the DRIs of potassium, calcium, and magnesium, expanding the potential for nutritional and health claims.

### 3.6. Practical Suggestions to Producers

#### 3.6.1. Multivariate Evaluation of the Mineral Composition of Experimental Treatments

**Figure 4:**
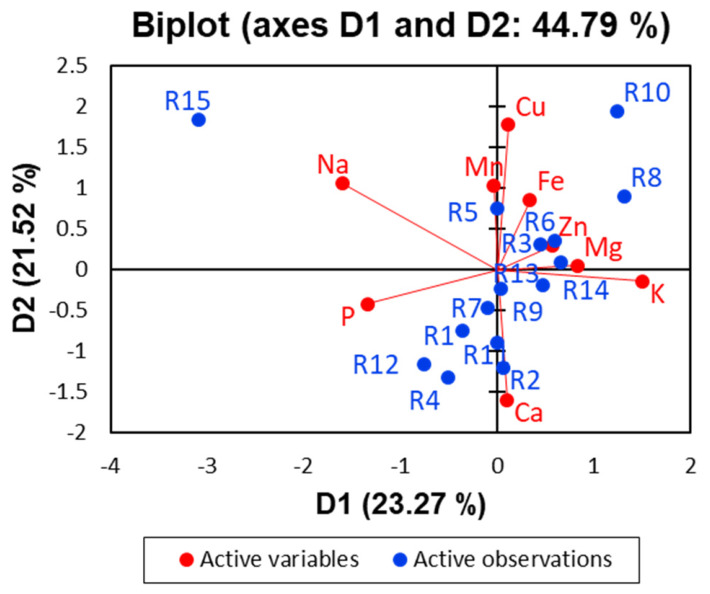
Visualisation of the relationship between experimental design treatments for the production of mineral fortified pimento-paste-stuffed olives and mineral contents using PCA biplot.

Multivariate analysis was used to visualise the relationships between experimental treatments (runs) and their corresponding mineral compositions. A principal components biplot (Figure 4) provided an overview of the main patterns. The Control (R15) was clearly separated from all fortified treatments, mainly due to its high sodium (Na) content. Most fortified treatments clustered near calcium (Ca) along the Dim1 axis, reflecting the strong influence of CaCl_2_. Potassium and magnesium also correlated with Dim1 and were key factors for separation along Dim2. Among naturally occurring elements, treatments R10 and R8 were associated with elevated micronutrient levels. Phosphorus contributed to separation along Dim1, especially for R12, R14, and other treatments on the left side of the biplot. Copper (Cu) contributed to Dim2, with an orientation opposite to calcium. This relationship may be explained by their similar affinity for stuffed-olive components and their tendency to form complexes with chlorophyll derivatives [29].

The dispersion pattern suggested a possible grouping of treatments based on mineral profiles. Cluster analysis confirmed the existence of four groups (Tables S1 and S2). The Control (R15, class 4) showed a very high sodium content. High calcium levels were observed in Class 1 (R1 and R4), while Class 3 (R3 and R8) showed the highest potassium content. Most treatments belonged to Class 2, which had moderate potassium and the lowest magnesium levels. From a practical perspective, Class 2 provides a balanced base for product development; however, producers may want to increase its magnesium levels to enhance nutritional claims and consumer appeal.

#### 3.6.2. Optimisation

**Figure 5:**
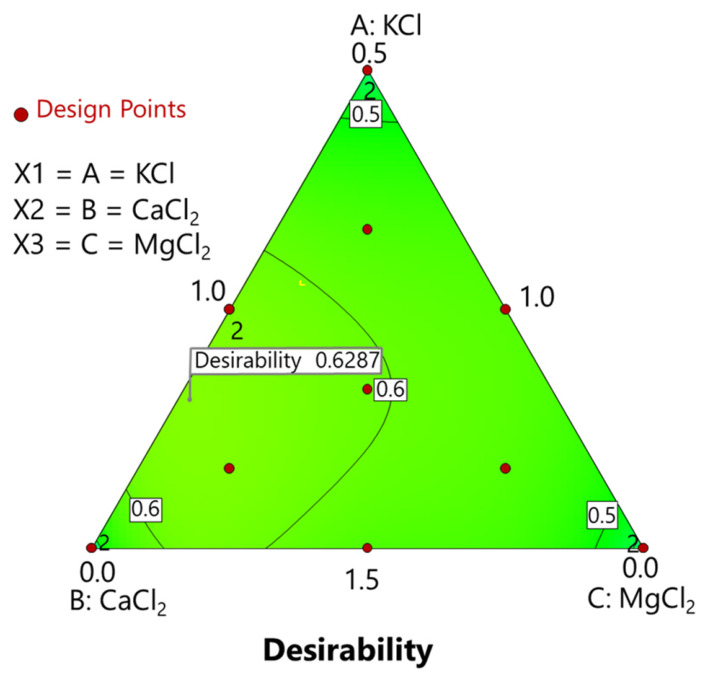
Optimisation of mineral chloride salt combinations using Response Surface Methodology (RSM). Suggested KCl, CaCl_2_, and MgCl_2_ concentrations, based on the established constraints (Table S3).

RSM was used to optimise salt combinations within potential constraints required by producers or consumers. In this case, the goal was to maximise both moisture content (to control product weight) and mineral concentrations (for the greatest health benefits) in Manzanilla pimento-stuffed olives (Figure 5). A detailed description of the constraints is provided in Table S3.

The optimisation process provided two possible solutions (Table 10). The concentrations of the fortifying salts were very similar, and their desirability was the same, although the programme selected the first one.

The best concentrations of the diverse salts under the constraints detailed in Table S3 were 1.189% KCl, 0.334% CaCl_2_, and 0.978% (Table 10), although the second solution was nearly as desirable.

This approach demonstrates how producers can use the developed RSM models to forecast outcomes across various salt formulations and evaluate their effects without additional experiments.

## 4. Discussion

Most studies in the literature highlight the importance of reducing sodium during fermentation, despite the potential risks involved [30]. Pires-Cabral et al. [31] assessed the effectiveness of seasoning *Cobrançosa* table olives in a brine with aromatic ingredients to mask the bitter taste caused by KCl when added to reduced-sodium fermentation brines. Olives fermented in a 4% NaCl + 4% KCl solution contained half the sodium but had higher levels of potassium and calcium and were more bitter than those fermented in NaCl alone. Seasoning the olives with a 4% NaCl + 4% KCl solution reduced bitterness and improved overall flavour and acceptance.

Eid et al. [32] developed an innovative Egyptian-style sweet-and-sour table olive by adding 3% sugar in two treatments (NaOH-treated olives and natural olives) during fermentation and packaging. The product displayed a unique profile, characterised by increased acidity and sweetness; however, most treatments still contained 7% salt.

Recent EU projects, such as “Development of innovative table olive products with low salt content and increased shelf life” (Olivia) [33], focus on fermentation. Its goal was to utilise alternative osmotic solvents to assist in olive dehydration, combined with an edible coating that preserved moisture, texture, and visual appeal.

In contrast, no prior studies have examined mineral fortification or sodium reduction during the packaging of pimento-paste-stuffed olives.

The storage brine of pimento-paste-stuffed olives (the raw material) showed a high sodium concentration to prevent spoilage while awaiting packaging, whereas the pitted olives and the stuffing material contained lower concentrations, with the pimento paste being relatively richer in minerals. This distribution is consistent with other studies describing the equilibrium between sodium and potassium in olives (and their moisture) and brines [15,16,21]. In contrast, calcium and magnesium were more strongly retained in the solid fractions, particularly in the stuffing material, while phosphorus was more abundant in brine. Trace elements such as iron, copper, manganese, and zinc remained predominantly associated with the flesh, exhibiting limited losses.

The distribution of minerals among the storage brine, olives, and stuffing material—reported here for the first time—highlighted distinct partitioning behaviours. Sodium and potassium approached equilibrium with the product moisture, whereas divalent cations, particularly calcium, showed a stronger association with olive and pimento-paste components. These patterns align with observation results in whole green *Manzanilla* olives [15] and directly brined *Aloreña de Málaga* [16].

In the stuffing material, Fe, Cu, Mn, and Zn showed lower *K_d_* values than in the olive or whole product, whereas Na, K, Ca, Mg, and P showed higher *K_d_* values in the entire product. Notably, only Ca exhibited a high *K_d_* when calculated on a paste-moisture basis, indicating a stronger association with the pimento-paste gel components. Conversely, Na, K, and P were nearly in equilibrium between the brine and the stuffing material’s moisture.

During the conditioning operation (size grading, pitting, or pitting and stuffing), Spanish-table olives undergo significant changes in the composition of the storage brine. However, the sodium concentration must be maintained within the range of 7.5–8.5% to prevent product spoilage, particularly by inhibiting the growth of *Propionibacterium*, which can cause the defect known as “zapateria” [34]. Consequently, table olives awaiting packaging, as well as those already pitted or stuffed, typically exhibit high sodium chloride levels that need to be reduced at packaging [5]. This circumstance prompted a detailed study of the desalting process [35], whose kinetics were modelled at laboratory scale and subsequently confirmed at pilot plant [36]. The findings of these studies were instrumental in establishing the operational conditions applied in the present work. The results obtained here are consistent with the trends reported in those studies. The desalting process caused a slight quality deterioration, mainly affecting colour and texture, although the most pronounced changes were observed in the mineral nutrient composition. Desalting effectively reduced sodium but also led to substantial losses of other minerals, confirming previous findings [15,16]. Although this process reduces sodium content, it might not be suitable for industrial applications due to mineral nutrient depletion and processing inefficiencies. Instead, the direct use of low-sodium packaging brines appears to be a more appropriate strategy for achieving salt reduction while preserving nutritional quality.

Fortification through the modified formulation significantly increases the potassium, calcium, and magnesium content of the final product while maintaining reduced sodium levels. After equilibration, sodium and potassium largely followed moisture equilibrium, whereas calcium exhibited stronger binding to olive and pimento-paste matrices, and magnesium remained weakly associated. Phosphorus again showed strong bindings, often leading to overestimated distribution coefficients. This strong association of minerals, particularly calcium, has also been reported as a key factor contributing to the texture improvement of various Iranian cultivars [37].

Linear modelling confirmed that sodium, potassium, and magnesium interacted weakly with the matrix, whereas calcium showed a greater affinity for the solid components. The influence of chloride salt mixtures on mineral distribution was generally minor, although interactions involving calcium and magnesium were detected. Importantly, predictive models relating brine composition to final mineral content proved effective, enabling future estimation of nutritional composition and %DRI [38] prior to processing. These findings are consistent with previous observations in *Manzanilla* [15] and *Aloreña de Málaga* olives [16].

Multivariate analysis has been widely used to characterisation table olives based on their mineral nutrient composition [3] as well as their fibre, sugars, and organic acid profiles [39]. In this study, multivariate and optimisation analysis further demonstrated the feasibility of tailoring mineral profiles through brine design, identifying distinct product classes and an optimal formulation that balances sodium reduction with mineral fortification. Overall, these results provide a practical framework for improving the nutritional quality of pimento-paste-stuffed olives through targeted brine formulation.

This study aligns with current strategies to reduce salt in foods while maintaining product safety. In this context, table olive fermentation can still be conducted at conventional salt levels—ensuring microbiological stability—provided that a subsequent desalting step is applied during packaging, as proposed here. This approach offers a practical compromise between safety and sodium reduction. Moreover, this strategy may be extended to other applications, such as the revalorisation of table olive by-products. In these cases, the relatively small volume of material facilitates desalting, making the process more manageable at an industrial level. Indeed, salt does not appear to be a limiting factor for the development of derived ingredients, as recently suggested by Rus-Fernández et al. [40,41]. Their proposed process, comprising conditioning, washing to reduce salt content, pressing, and drying, demonstrated the feasibility of producing functional ingredients from table olives. The freeze-dried powder showed the highest phenolic content, exceeding 2500 mg (GAE/kg), and exhibited antioxidant properties, effectively reducing lipid oxidation to below 2 MDA/kg. The residual salt was not a drawback but acted as an effective flavour enhancer, further supporting the potential of these products.

In contrast, Papapostolou et al. [42] also examined, employing RSM, the application of olive oil flavoured with essential oils as a preservation system for reduced-sodium Spanish-style table olives (cv. *Chalkidiki*). Oregano essential oil showed a strong antimicrobial effect against pathogens, whereas lemon balm and bay laurel essential oils were more effective against yeasts. The results highlight the potential of flavoured olive oil as an alternative preservation method for customised low-sodium products, particularly in settings with limited technological resources, where simple, cost-effective solutions are required.

By contrast, the approach proposed in the present study may have broader industrial applicability, as it relies on pasteurisation, a widely implemented preservation method in current table olive packaging. Nevertheless, both strategies can be considered complementary. The use of essential oils could be adapted for seasoning purposes at lower concentrations—compatible with sensory acceptance rather than for microbial Control alone. Recent initiatives such as the OLIPACK project [43] further reflect the ongoing interest in low-salt table olives. This project focuses on innovative packaging systems that incorporate natural antimicrobial agents derived from olive wastewater and biodegradable polymers from olive tree residues. While such approaches aim to enhance sustainability and shelf life, their effectiveness as a standalone preservation system remains uncertain under strict pH and NaCl conditions [44].

Alternative strategies, such as modified atmosphere packaging, have also shown promising results. Tzamourani et al. [45] demonstrated that Spanish-style green olives can be stored for extended periods under reduced salt conditions, maintaining microbial stability with only moderate changes in pH. This approach can also be adapted for mineral-fortified olive oil packaged in flexible formats.

Overall, these developments highlight the strong interest from both research initiatives and policy frameworks in promoting reduced-salt products. Importantly, this trend is also driven by consumers’ demand. Paltaki et al. [46] reported a clear preference for reduced-sodium table olives and a high willingness to purchase them. This convergence of consumer expectations, industrial feasibility, and scientific innovation supports the development of new low-salt formulations. In this context, the present study provides practical tools to help producers design products that meet both nutritional targets and market demand.

## 5. Conclusions

This study is the first to evaluate the combined effects of sodium reduction and the addition of potassium, calcium, and magnesium in Spanish-style pimento-paste-stuffed olives using Response Surface Methodology (RSM). Mineral determination in olives and brines showed that sodium is generally more concentrated in brines (29.7 g/kg) than in pitted olives (20.1 g/kg), the stuffing material (26.5 g/kg), or the whole product (21.2 g/kg). This distribution may mislead consumers and nutritionists regarding the actual sodium content of the product.

Desalting effectively reduced sodium levels (~65%) but also caused substantial losses in potassium (~65%), calcium (~45%), magnesium (~61%), and other nutrients. This indicates that fermentation and conditioning processes can have a negative nutritional impact, decreasing the overall mineral value of table olives and highlighting the need for mineral adjustment during packaging.

Distribution coefficients, a recently developed index to assess the partitioning of minerals between brine and olives, have proven to be a valuable tool for evaluating mineral-matrix interactions in olives, stuffing material, and stuffed olives. These coefficients support the strong association of calcium and phosphorus with olive organic compounds, as reflected by K*d* values markedly higher than 1. In contrast, potassium (predominantly) and magnesium are mainly solubilized in the aqueous phase.

The experimental packaging formulation resulted in significantly healthier products, with reduced sodium (7 g/kg, 30% DRI) but increased levels of potassium (up to 5.4 g/kg; 27% DRI), calcium (~4.6 g/kg; 58% DRI), and magnesium (~1.8 g/kg; 32% DRI). Furthermore, the predictive response surface models enabled accurate estimation of mineral composition, dietary contributions, and process optimisation.

Overall, these findings provide both mechanistic insight and practical tools for designing tailored low-sodium and fortification strategies, supporting the production of Spanish-style stuffed olives with reduced sodium and enhanced nutritional value, while also offering approaches applicable to a broader range of fermented products.

## Figures and Tables

**Table 1:** Simplex lattice mixture experimental design to study pimento-paste-stuffed olive fortification.

Treatment	[KCl]	[CaCl_2_]	[MgCl_2_]
1	0.5	1.0	1.0
2	1.5	1.0	0.0
3	1.5	0.0	1.0
4	0.5	1.0	1.0
5	1.0	0.5	1.0
6	1.5	0.5	0.5
7	0.83	0.83	0.83
8	1.5	0.0	1.0
9	1.5	1.0	0.0
10	1.33	0.33	0.83
11	1.33	0.83	0.33
12	1.17	0.67	0.67
13	1.0	0.5	1.0
14	1.0	1.0	0.5
15 (Control)	0.0	0.0	0.0

Notes: Concentrations (%) expected in the packaged products after equilibrium. Salt concentrations in the initial brines were adjusted based on the salt’s hydration levels and the ratio of stuffed olives to brine in the containers. Control was packaged in the usual industrial conditions (5% salt). Salt concentrations in the treatments were 2.5% NaCl plus the design treatment (2.5%), for a total of 5%.

**Table 2:** Nutrient mineral contents in the whole pimento-paste-stuffed olives and their components (olive and pimento-paste), as well as mineral losses during desalting.

Nutrient	Mineral Contents in Stored Stuffed Olives and Their Components						Mineral Content in Desalted Stuffed Olives and Their Components						Mineral Losses (%)		
	Whole Product	Pitted Olives	Stuffing Material	Brine	Pooled SE		Whole Product	Pitted Olives	Stuffing Material	Desalting Solution	Pooled SE		Whole Product	Pitted Olives	Stuffing Material
Na	21,198	20,069	26,542	29,656	133		7340	6817	9260	9938	85		65.37	66.03	65.11
K	659	647	858	884	8		232	213	325	325	1		64.80	67.08	62.12
Ca	2813	2339	3227	1653	33		1555	1403	1670	611	9		44.72	40.02	48.25
Mg	194	181	233	212	3		76.5	73.4	96.1	75.8	0.5		60.57	59.45	58.76
Fe	29.1	27.3	22.7	3.4	1.2		24.9	30.1	16.7	2.5	1		14.43	NL	26.43
Cu	1.3	1.69	1.03	0.66	0.06		1.08	1.39	0.87	0.72	0.06		16.92	17.75	15.53
Mn	0.28	0.48	0.33	0.25	0.05		0.27	0.3	0.21	0.23	0.02		3.57	37.50	36.36
Zn	2.56	2.58	2	1.52	0.1		2.08	2.46	1.82	1.16	0.11		18.75	4.65	9.00
P	115	120	129	132	2		74.8	75.2	74.8	36.9	0.3		34.96	37.33	42.02

Notes: Concentrations in mg/kg; SE, standard error; NL, no loss detected.

**Table 3:** Distribution coefficients of minerals in the stored whole pimento-paste-stuffed olives, their components (olive and pimento paste), and their respective moisture contents.

Nutrient	Distribution Coefficients in Stored Stuffed Olives and Their Components				Distribution Coefficients in Stored Stuffed Olives and Their Components Based on Moisture		
	Whole Product	Pitted Olives	Stuffing Material		Whole Product	Pitted Olives	Stuffing Material
Na	0.7148 (0.0037)	0.6768 (0.0050)	0.8951 (0.0108)		0.9797 (0.0052)	0.9881 (0.0073)	1.0300 (0.0124)
K	0.7462 (0.0047)	0.7323 (0.0136)	0.9702 (0.0107)		1.0227 (0.0065)	1.0692 (0.0199)	1.1165 (0.0123)
Ca	1.7012 (0.0254)	1.4147 (0.0081)	1.9516 (0.0175)		2.3316 (0.0348)	2.0655 (0.0119)	2.2457 (0.0201)
Mg	0.9153 (0.0271)	0.8523 (0.0103)	1.0971 (0.0112)		1.2546 (0.0372)	1.2444 (0.0151)	1.2625 (0.0129)
Fe	8.5230 (0.5966)	7.9894 (0.2399)	6.6214 (0.1020)		11.6908 (0.8177)	11.6651 (0.3502)	7.6196 (0.1174)
Cu	1.9788 (0.0677)	2.5788 (0.1623)	1.565 (0.0437)		2.7121 (0.0927)	3.7652 (0.2369)	1.8009 (0.0503)
Mn	1.1109 (0.1466)	1.9238 (0.2334)	1.2832 (0.2422)		1.5226 (0.0209)	2.8089 (0.3407)	1.4766 (0.2787)
Zn	1.6761 (0.1015)	1.6898 (0.0301)	1.3137 (0.0287)		2.2973 (0.1392)	2.4672 (0.0440)	1.5118 (0.0330)
P	0.8723 (0.0171)	0.9102 (0.0238)	0.9795 (0.0172)		1.1956 (0.0234)	1.3289 (0.0348)	1.1272 (0.0198)

Notes: Standard error in parentheses. Moisture of whole stuffed olives, 72.96%; pitted olives (olives without stone), 68.49%; pimento paste, 86.90%.

**Table 4:** Mineral concentrations (mg/kg) and moisture (%, *w*/*w*) in the experimental design treatments of the fortified pimento-paste-stuffed olives and the Control.

Treatment	[Na]	[K]	[Ca]	[Mg]	[Fe]	[Cu]	[Mn]	[Zn]	[P]	Stuffed Olive Moisture
1	7101 (99)	1958 (11)	4564 (18)	1761 (3)	22.72 (0.47)	0.80 (0.12)	0.256 (0.018)	2.01 (0.14)	57.40 (1.29)	73.6908 (0.0004)
2	7134 (93)	5335 (48)	4584 (16)	50.12 (0.47)	23.28 (0.75)	0.72 (0.06)	0.226 (0.017)	1.74 (0.06)	54.29 (0.32)	75.6253 (0.0003)
3	7159 (74)	5279 (9)	1141 (9)	1776 (17)	23.51 (0.67)	0.81 (0.11)	0.246 (0.029)	1.77 (0.09)	55.84 (1.38)	75.4295 (0.0002)
4	7128 (51)	1867 (7)	4583 (5)	1771 (10)	22.32 (0.30)	0.62 (0.05)	0.262 (0.030)	1.94 (0.18)	56.82 (0.96)	74.1571 (0.0008)
5	7164 (42)	3667 (28)	2771 (19)	1781 (18)	23.20 (0.28)	1.06 (0.07)	0.288 (0.007)	1.89 (0.10)	57.69 (1.36)	75.2341 (0.0007)
6	7223 (73)	5359 (29)	2802 (14)	919 (7)	23.30 (0.61)	0.86 (0.19)	0.280 (0.010)	1.97 (0.16)	55.37 (1.30)	73.8219 (0.0014)
7	7283 (45)	3008 (10)	3932 (44)	1468 (17)	23.13 (0.41)	0.88 (0.01)	0.236 (0.011)	1.97 (0.16)	54.33 (1.37)	74.6851 (0.0009)
8	7076 (32)	5363 (5)	1171 (8)	1779 (15)	22.52 (0.23)	1.09 (0.08)	0.200 (0.015)	1.92 (0.09)	52.68 (1.02)	74.8559 (0.0006)
9	7083 (32)	5336 (22)	4524 (20)	49.53 (0.47)	22.62 (0.95)	0.97 (0.06)	0.274 (0.024)	1.87 (0.08)	55.98 (1.18)	75.0357 (0.0002)
10	7134 (71)	4925 (11)	2374 (20)	1485 (5)	23.63 (0.67)	1.16 (0.06)	0.329 (0.021)	2.02 (0.13)	53.82 (0.93)	74.2239 (0.0028)
11	7099 (88)	4863 (20)	3972 (41)	632 (1)	21.30 (0.03)	0.95 (0.08)	0.195 (0.055)	1.96 (0.17)	56.43 (0.83)	74.2336 (0.0032)
12	7150 (34)	4119 (23)	3396 (12)	1165 (8)	19.86 (0.18)	0.82 (0.10)	0.250 (0.010)	1.88 (0.06)	58.84 (0.60)	74.7587 (0.0004)
13	7075 (38)	3798 (40)	2830 (20)	1797 (9)	20.13 (0.39)	1.01 (0.08)	0.225 (0.012)	1.89 (0.09)	53.60 (0.66)	74.0339 (0.0008)
14	7061 (52)	3721 (26)	4605 (17)	921 (6)	22.13 (0.63)	1.02 (0.03)	0.265 (0.023)	1.97 (0.15)	52.73 (0.78)	74.1254 (0.0018)
15 (Control)	14,216 (28)	118 (2)	1137 (24)	45.60 (0.12)	22.51 (0.31)	1.14 (0.05)	0.266 (0.014)	1.82 (0.09)	57.92 (0.60)	75.0587 (0.0021)

Notes: Standard error in parentheses.

**Table 5:** Mineral concentrations (mg/kg) in the experimental design treatments of the fortified pimento-paste-stuffed olives and Control, based on the stuffed olive moisture, and their brines.

Treatment	Contents Based on the Stuffed Olive Moisture						Brines				
	[Na]	[K]	[Ca]	[Mg]	[P]		[Na]	[K]	[Ca]	[Mg]	[P]
1	9636 (128)	2657 (13)	6194 (21)	2389 (3)	77.90 (1.74)		9697 (21)	2596 (11)	3593 (13)	2236 (10)	33.86 (0.25)
2	9433 (127)	7054 (63)	6062 (18)	66.27 (0.58)	71.78 (0.46)		9918 (156)	7210 (26)	3588 (10)	60.79 (0.06)	33.63 (0.17)
3	9490 (101)	6998 (13)	1513 (11)	2354 (23)	74.03 (1.82)		9838 (26)	7204 (15)	584 (1)	2271 (7)	33.84 (0.11)
4	9613 (76)	2518 (9)	6181 (16)	2388 (15)	76.62 (1.19)		9983 (56)	2587 (13)	3591 (6)	2236 (9)	35.70 (0.70)
5	9522 (43)	4874 (41)	3683 (30)	2368 (21)	76.67 (1.75)		9925 (114)	5017 (22)	2115 (3)	2249 (12)	36.57 (0.33)
6	9784 (78)	7259 (27)	3796 (13)	1245 (8)	75.01 (1.90)		9840 (18)	7245 (29)	2048 (4)	1126 (4)	35.68 (0.85)
7	9751 (75)	4028 (20)	5264 (52)	1966 (20)	72.75 (1.94)		9783 (18)	4113 (18)	3088 (2)	1834 (5)	34.98 (0.63)
8	9452 (49)	7164 (2)	1564 (9)	2377 (22)	70.38 (1.42)		9813 (30)	7214 (23	516 (2)	2276 (4)	36.48 (0.55)
9	9439 (39)	7112 (33)	6030 (25)	66.01 (0.63)	74.60 (1.59)		9821 (81)	7213 (35)	3611 (3)	57.72 (0.20)	36.50 (0.22)
10	9613 (129)	6636 (37)	3198 (23)	2001 (9)	72.52 (1.38)		9737 (65)	6680 (28)	1635 (4)	1860 (3)	36.78 (1.10)
11	9564 (168)	6551 (34)	5352 (87)	851 (6)	76.03 (1.45)		9779 (32)	6687 (16)	3022 (2)	751 (5)	37.26 (0.59)
12	9564 (51)	5509 (32)	4543 (19)	1558 (9)	78.71 (0.83)		9709 (14)	5638 (23)	2610 (3)	1442 (2)	35.52 (0.95)
13	9556 (58)	5130 (48)	3822 (32)	2428 (11)	72.40 (0.79)		9826 (26)	5127 (36)	2112 (7)	2291 (13)	34.48 (1.13)
14	9526 (93)	5020 (41)	6212 (3)	1242 (12)	71.14 (1.20)		9731 (67)	5036 (36)	3606 (4)	1170 (9)	36.70 (0.60)
15 (Control)	18,941 (97)	156.55 (2.72)	1516 (37)	60.75 (0.40)	77.17 (0.82)		18,948 (144)	162.18 (2.32)	519 (2)	56.49 (0.09)	33.82 (0.63)

Note: Standard error in parentheses. Micronutrients in brine were below detection limits. So, they were not subjected to the distribution coefficient study.

**Table 6:** Distribution coefficients of the major mineral nutrients in pimento-paste-stuffed olive, based on the whole product and its moisture.

Treatment	*K_d_* Based on Stuffed Olives Versus Brines						*K_d_* Based on Stuffed Olive Moistures Versus Brine						Stuffed Olive Moisture (%, *w*/*v*)
	[Na]	[K]	[Ca]	[Mg]	[P]		[Na]	[K]	[Ca]	[Mg]	[P]		
1	0.7322 (0.0093)	0.7544 (0.0073)	1.2703 (0.0078)	0.7873 (0.0049)	1.6959 (0.0490)		0.9937 (0.0119)	1.0237 (0.0092)	1.7238 (0.0104)	1.0683 (0.0063)	2.3013 (0.0658)		73.6908 (0.0004)
2	0.7195 (0.0120)	0.7399 (0.0080)	1.2778 (0.0065)	0.8245 (0.0084)	1.6146 (0.0158)		0.9514 (0.0165)	0.9784 (0.0104)	1.6897 (0.0080)	1.0902 (0.0105)	2.1349 (0.0221)		75.6253 (0.0003)
3	0.7277 (0.0095)	0.7327 (0.0013)	1.9546 (0.0169)	0.7821 (0.0089)	1.6504 (0.0431)		0.9647 (0.0029)	0.9714 (0.0014)	2.5913 (0.0214)	1.0368 (0.0122)	2.1880 (0.0571)		75.4295 (0.0002)
4	0.7141 (0.0082)	0.7218 (0.0060)	1.2764 (0.0032)	0.7920 (0.0030)	1.5926 (0.0319)		0.9630 (0.0113)	0.9733 (0.0076)	1.7212 (0.0064)	1.0679 (0.0035)	2.1476 (0.0331)		74.1571 (0.0008)
5	0.7220 (0.0100)	0.7308 (0.0042)	1.3102 (0.0077)	0.7919 (0.0098)	1.5769 (0.0267)		0.9597 (0.0124)	0.9714 (0.0067)	1.7415 (0.0122)	1.0526 (0.0115)	2.0960 (0.1042)		75.2341 (0.0007)
6	0.7340 (0.0081)	0.7397 (0.0054)	1.3684 (0.0080)	0.8165 (0.0066)	1.5553 (0.0734)		0.9943 (0.0092)	1.0020 (0.0070)	1.8536 (0.0090)	1.1060 (0.0082)	2.1073 (0.0087)		73.8219 (0.0014)
7	0.7444 (0.0059)	0.7314 (0.0016)	1.2729 (0.0143)	0.8007 (0.0069)	1.5527 (0.0121)		0.9968 (0.0093)	0.9793 (0.0022)	1.7043 (0.0165)	1.0720 (0.0075)	2.0790 (0.0659)		74.6851 (0.0009)
8	0.7211 (0.0043)	0.7433 (0.0029)	2.2703 (0.0088)	0.7817 (0.0063)	1.4455 (0.0477)		0.9633 (0.0059	0.9930 (0.0031)	3.0329 (0.0091	1.0443 (0.0095)	1.9312 (0.0403)		74.8559 (0.0006)
9	0.7212 (0.0041)	0.7399 (0.0063)	1.2531 (0.0048)	0.8581 (0.0059)	1.5335 (0.0298)		0.9611 (0.0055)	0.9861 (0.0087)	1.6700 (0.058)	1.1435 (0.0078)	2.0437 (0.0487)		75.0357 (0.0002)
10	0.7328 (0.0122)	0.7374 (0.0046)	1.4515 (0.0151)	0.7981 (0.0035)	1.4656 (0.0431)		0.9875 (0.0199)	0.9935 (0.0067)	1.9556 (0.0158)	1.0753 (0.0034)	1.9741 (0.0403)		74.2239 (0.0028)
11	0.7259 (0.0094)	0.7272 (0.0043)	1.3146 (0.0144)	0.8411 (0.0060)	1.5145 (0.0025)		0.9781 (0.0182)	0.9797 (0.0071)	1.7712 (0.0297)	1.1332 (0.0147)	2.0403 (0.0487)		74.2336 (0.0032)
12	0.7364 (0.0034)	0.7306 (0.0069)	1.3013 (0.0034)	0.8075 (0.0055)	1.6598 (0.0611)		0.9851 (0.0049)	0.9773 (0.0095)	1.7406 (0.0056)	1.0801 (0.0069)	2.2203 (0.0828)		74.7587 (0.0004)
13	0.7199 (0.0028)	0.7409 (0.0127)	1.3396 (0.0127)	0.7847 (0.0066)	1.5580 (0.0570)		0.9725 (0.0040)	1.0007 (0.0159)	1.8095 (0.0190)	1.0600 (0.0074)	2.1044 (0.0754)		74.0339 (0.0008)
14	0.7257 (0.0099)	0.7389 (0.0064)	1.2769 (0.0058)	0.7870 (0.0101)	1.4372 (0.0222)		0.9790 (0.0146)	0.9969 (0.0118)	1.7226 (0.0020)	1.0618 (0.0162)	1.9391 (0.0367)		74.1254 (0.0018)
15 (Control)	0.7504 (0.0060)	0.7251 (0.0219)	2.1896 (0.0404)	0.8072 (0.0030)	1.7135 (0.0232)		0.9998 (0.0118)	0.9661 (0.0298)	2.9177 (0.0653)	1.0755 (0.0081)	2.2827 (0.0227)		75.0587 (0.0021)

Notes: Standard error in parentheses.

**Table 7:** Regression parameters of the mineral contents (mg/kg) in pimento-paste-stuffed olives and brine (mg/kg), based on the whole product or its moisture.

Model/Summary		Na	K	Ca	Mg
Concentration in whole product vs. brine					
Full model	Adjusted R^2^	0.9931	0.9986	0.9975	0.9988
	*p*-value	<0.0001	<0.0001	<0.0001	<0.0001
Regression summary	β	0.9966	0.9993	0.9988	0.9994
	SE of β	0.0125	0.0057	0.0075	0.0052
	Intercept	−451.31	−7.43	516.86	15.511
	SE	103.55	23.99	22.46	6.816
	*p*-value	<0.0001	0.7583	<0.0001	0.0280
	Slope	0.773	0.7378	1.1213	0.7833
	SE	0.0097	0.0042	0.0084	0.004
	*p*-value	<0.0001	<0.0001	<0.0001	<0.0001
Concentration in whole product moisture vs. brine					
Full model	Adjusted R^2^	0.9931	0.9980	0.9965	0.9985
	*p*-value	<0.0001	<0.0001	<0.0001	<0.0001
Regression summary	β	0.9951	0.9990	0.9983	0.9993
	SE of β	0.0151	0.0068	0.0089	0.0058
	Intercept	−462.28	15.69	687.13	22.98
	SE	165.38	38.27	36.03	10.37
	*p*-value	0.0077	0.6837	<0.0001	0.0320
	Slope	1.022	0.9837	1.5073	1.050
	SE	0.016	0.0067	0.0135	0.0061
	*p*-value	<0.0001	<0.0001	<0.0001	<0.0001

Notes: β, standard coefficient; SE, standard error.

**Table 8:** Coded RSM coefficients of the models relating the calcium and magnesium distribution coefficients, based on whole product and moisture, as a function of the KCl, CaCl_2_, and MgCl_2_ contents in the packaging brines.

Component	Coefficient Estimate	df	Standard Error	95% CI Low	95% CI High	VIF
Model relating salt mixture concentrations with *K*_dCa_						
KCl	1.73	1	0.0859	1.53	1.92	1.62
CaCl_2_	2.79	1	0.0859	2.6	2.99	1.62
MgCl_2_	1.69	1	0.0858	1.49	1.89	1.5
KCl · CaCl_2_	−1.95	1	0.4062	−2.89	−1.01	1.76
KCl · MgCl_2_	0.27	1	0.4869	−0.8529	1.39	1.55
CaCl_2_ · MgCl_2_	−1.52	1	0.4869	−2.64	−0.3955	1.55
Model relating salt mixture concentrations with *K*_dMg_						
KCl	1.06	1	0.0113	1.04	1.09	1.09
CaCl_2_	1.05	1	0.0113	1.03	1.08	1.09
MgCl_2_	1.12	1	0.0115	1.1	1.15	1.06

Notes: 95% CI Low, Lowest Confidence Interval at 95% confidence. 95% CI High, Highest Confidence Interval at 95% confidence. VIF, Variance Inflation Factor.

**Table 9:** Coded coefficients of models relating the mineral contents in the pimento-paste-stuffed olives (olive plus pimento-paste) as a function of the salt mixture concentrations in the packaging brines.

Component	Coefficient Estimate	df	Standard Error	95% CI Low	95% CI High	VIF
Model relating K content in pulp with salt mixture in packaging brine						
KCl	1940.32	1	48.22	1834.2	2046.44	1.09
CaCl_2_	5377.55	1	48.22	5271.42	5483.67	1.09
MgCl_2_	5360.21	1	49.33	5251.63	5468.79	1.06
Model relating Ca content in the product to the salt mixture in the packaging brine						
KCl	4550.35	1	28.22	4488.23	4612.47	1.09
CaCl_2_	1143.81	1	28.22	1081.69	1205.93	1.09
MgCl_2_	4552.43	1	28.88	4488.87	4615.98	1.06
Model relating Mg content in the product to the salt mixture in the packaging brine						
KCl	1764.39	1	6.59	1748.8	1779.98	1.62
CaCl_2_	1778.01	1	6.59	1762.42	1793.6	1.62
MgCl_2_	52.11	1	6.59	36.52	67.7	1.5
KCl · CaCl_2_	68.31	1	32.76	−9.15	145.77	1.94
KCl · MgCl_2_	57.21	1	41.64	−41.25	155.68	1.93
CaCl_2_ · MgCl_2_	37.84	1	41.64	−60.63	136.3	1.93
KCl · CaCl_2_ · MgCl_2_	−1184.86	1	288.49	−1867.04	−502.69	2.22

Notes: 95% CI Low, Lowest Confidence Interval at 95% confidence. 95% CI High, Highest Confidence Interval at 95% confidence. VIF, Variance Inflation Factor.

**Table 10:** RSM optimisation process. Recommended and selected concentrations of the KCl, CaCl_2_, and MgCl_2_ for the packaging brines, as well as predicted values expected for the constrained parameters in the resulting fortified pimento-paste-stuffed olives.

Solutions		
Constrain	1 (Selected)	2
KCl	1.189	1.169
CaCl_2_	0.334	0.331
MgCl_2_	0.978	1.000
Moisture (%)	74.74	74.791
Na	7139.805	7140.200
K	4307.328	4238.109
Ca	2279.956	2273.075
Mg	1745.016	1788.633
Fe	22.508	22.500
Cu	0.964	0.963
Zn	1.898	1.892
Mn	0.253	0.253
P	55.176	55.200
Na % (DRI)	29.749	29.751
K % (DRI)	21.537	21.191
Ca % (DRI)	28.499	28.413
Mg % (DRI)	46.534	47.697
Desirability	0.629	0.629

Note: Concentrations of salt mixtures and moisture in percentages (%); Mineral concentrations (in mg/kg); DRI, daily reference intake.

## Data Availability

The original contributions presented in the study are included in the article; further inquiries can be directed to the corresponding author.

## Supplementary Materials

The following supporting information can be downloaded at: https://www.mdpi.com/article/10.3390/foods15101658/s1. Table S1: Distribution into classes of the experimental treatments of fortified pimento-paste-stuffed olives, based on their mineral content; Table S2: Mineral characterization of the classes resulting from the clustering analysis of the experimental treatments of fortified pimento-paste-stuffed olives; Table S3: Constrains applied for the optimization process by RSM, based on the experimental design conditions and ranges of mineral and DRI (%) responses on the fortified pimento-paste stuffed olives.
