# Supplementary material for: Mineral-Fortified and Sodium-Reduced Pimento-Paste-Stuffed Spanish-Style Manzanilla Olives
Source: Foods. 2026 May 9;15(10):1658. doi: 10.3390/foods15101658 (PMC13205983; doi:10.3390/foods15101658)
Supplement: Supplementary file 1 [file foods-15-01658-s001.zip › foods-4240915-supplementary.pdf]

Supplementary material to

Mineral-Fortified and Sodium-Reduced Pimento-Paste-Stuffed Spanish-Style *Manzanilla* Olives

Table S1. Distribution into classes of the experimental treatments of fortified pimento-paste-stuffed olives, based on their mineral content.

Class	1	2	3	4
Objects	2	10	2	1
Sum of weights	2	10	2	1
Within-class variance	4714.776	1865709.101	7419.992	0.000
Minimum distance to centroid	48.553	373.237	60.910	0.000
Average distance to centroid	48.553	1237.037	60.910	0.000
Maximum distance to centroid	48.553	1677.530	60.910	0.000
	R1	R2	R3	R15
	R4	R5	R8	
		R6		
		R7		
		R9		
		R10		
		R11		
		R12		
		R13		
		R14		

Table S2. Mineral characterization of the classes resulting from the clustering analysis of the experimental treatments of fortified pimento-paste-stuffed olives.

	Classes differentiated by cluster analysis			
Nutrient	Class 1	Class 2	Class 3	Class 4
Na	7114.733	7140.360	7117.033	14216.367
K	1912.617	4413.097	5320.550	117.500
Ca	4574.017	3578.930	1155.867	1137.300
Mg	1765.833	1026.783	1777.567	45.633
Fe	22.564	22.258	23.014	22.509
Cu	0.706	0.947	0.951	1.141
Mn	0.259	0.257	0.223	0.266
Zn	1.977	1.890	1.843	1.818
P	57.112	55.306	54.257	57.920

Notes: Mineral concentrations in mg/kg stuffed olive (olive plus pimento-paste). Runs included in each class. Class 1: R1 and R4. Class 2: R2, R5, R6, R7, R9, R10, R11, R12, R13 and R14.

Table S3. Constrains applied for the optimization process by RSM, based on the experimental design conditions and ranges of mineral and DRI(%) responses on the fortified pimento-paste-stuffed olives.

Name	Goal	Lower limit	Upper limit	Lower weight	Upper weight	Importance
KCl	is in range	0.5	1.5	1	1	3
$CaCl_2$	is in range	0	1	1	1	3
$MgCl_2$	is in range	0	1	1	1	3
Moisture	maximize	73.6908	75.6253	1	1	3
Na	minimize	7060.75	7282.57	1	1	3
K	maximize	1867.3	5362.57	1	1	3
Ca	maximize	1141.16	4604.57	1	1	3
Mg	maximize	49.5315	1797.33	1	1	3
Fe	maximize	19.857	23.63	1	1	3
Cu	maximize	0.615	1.16433	1	1	3
Zn	maximize	1.7421	2.0175	1	1	3
Mn	maximize	0.194667	0.329	1	1	3
P	maximize	52.679	58.8447	1	1	3
Na % (DRI)	minimize	29.4198	30.344	1	1	3
K % (DRI)	maximize	9.33651	26.8129	1	1	3
Ca % (DRI)	maximize	10.8937	20.9557	1	1	3
Mg % (DRI)	maximize	1.32084	47.9287	1	1	3

Note: Concentrations of salt mixtures in % (w/w); Moisture, in percentage; mineral concentrations (in mg/kg); DRI, daily reference intake.
